# The future development of intensive care quality indicators – a methods paper

**DOI:** 10.3205/000285

**Published:** 2020-10-30

**Authors:** Oliver Kumpf, Monika Nothacker, Jan Braun, Elke Muhl

**Affiliations:** 1 Department of Anaesthesiology and Intensive Care Medicine, Campus Charité Mitte and Campus Virchow-Klinikum, Charité – Universitätsmedizin Berlin, corporate member of Freie Universität Berlin, Humboldt-Universität zu Berlin, and Berlin Institute of Health, Berlin, Germany; 2 National Steering Committee Peer Review, German Interdisciplinary Association for Intensive Care and Emergency Medicine (DIVI), Berlin, Germany; 3 AWMF-Institute for Medical Knowledge Management c/o Philipps-Universität, Marburg, Germany; 4 Department of Anaesthesiology, Intensive Care Medicine and Pain Therapy, Martin-Luther-Krankenhaus, Berlin, Germany; 5 Groß Grönau, Germany

## Abstract

**Introduction:** Medical quality indicators (QI) are important tools in the evaluation of medical quality. Their development is subject to specific methodological requirements, which include practical applicability. This is especially true for intensive care medicine with its complex processes and their interactions. This methods paper presents the status quo and shows necessary methodological developments for intensive care QI. For this purpose, a cooperation with the Association of the Scientific Medical Societies’ Institute for Medical Knowledge Management (AWMF-IMWi) was established.

**Methodology:** Review of published German manuals for QI development from guidelines and narrative review of quality indicators with a focus on evidence and consensus-based guideline recommendations. Future methodological adaptations of indicator development for improved operationalization, measurability and pilot testing are presented, and a development process is proposed.

**Results:** The development of intensive care quality indicators in Germany is based on an established process. In the future, additional evaluation criteria (QUALIFY criteria) will be applied to assess the evidence base. In addition, a continuous exchange between the national steering committee of the DIVI responsible for QI development and guideline development groups involved in intensive care medicine is planned.

**Conclusion:** Intensive care quality indicators will have to meet improved methodological requirements in the future by means of an improved development process. Future QI development is intended to improve the structure of the development process, with a focus on scientific evidence and a link to guideline projects. This is intended to achieve the goal of a broad application of QI and to further evaluate its relevance for patient outcome and performance of institutions.

## Zusammenfassung

**Einleitung:** Medizinische Qualitätsindikatoren (QI) stellen ein wichtiges Werkzeug bei der Betrachtung medizinischer Qualität dar. Ihre Entwicklung unterliegt methodischen Anforderungen, die auch ihre praktische Anwendbarkeit einschließen. Dies gilt insbesondere für die Intensivmedizin mit ihren komplexen Prozessen und Interaktionen. Dieses Methodenpapier dient der Darstellung des Status quo und zeigt erforderliche methodische Weiterentwicklungen intensivmedizinischer QI auf. Dazu erfolgte eine Kooperation mit dem Institut für Medizinisches Wissensmanagement der Arbeitsgemeinschaft der Wissenschaftlichen Medizinischen Fachgesellschaften (AWMF-IMWi).

**Methodik:** Rückgriff auf publizierte deutsche Manuale zur QI-Ableitung aus Leitlinien und narratives Review zu Qualitätsindikatoren mit Fokus auf die Beziehung zu evidenz- und konsensbasierten Leitlinienempfehlungen. Die notwendigen methodischen Anpassungen der Indikatorentwicklung wie Operationalisierbarkeit, Messbarkeit und Pilotierung werden dargestellt, und ein Vorschlag für einen Entwicklungsprozess wird erarbeitet.

**Ergebnisse:** Die Entwicklung intensivmedizinischer Qualitätsindikatoren in Deutschland geschieht in einem etablierten Verfahren. Dieses soll künftig durch die Aufnahme zusätzlicher Bewertungskriterien (QUALIFY-Kriterien) u.a. zur Beurteilung der Evidenzbasierung erweitert werden. Ergänzend ist ein kontinuierlicher Austausch der für die QI verantwortlichen nationalen Steuerungsgruppe der DIVI mit den Leitliniengruppen geplant, die intensivmedizinische Fragestellungen bearbeiten.

**Schlussfolgerung:** Intensivmedizinische Qualitätsindikatoren können durch einen verbesserten Entwicklungsprozess in Zukunft gestiegenen methodischen Anforderungen genügen. Die beschriebenen Weiterentwicklungen sollen den Erstellungsprozess besser strukturieren, wissenschaftlicher gestalten und in Zukunft mit Leitlinienprojekten vernetzen. Dadurch soll das Ziel einer flächendeckenden Anwendung der QI erreicht werden und schließlich ihre Relevanz für das Outcome bezogen auf Patienten und Einrichtungen geprüft werden.

## 1 Introduction

The development of intensive care quality indicators (QI) in Germany was initiated by a working group within the German Society for Anaesthesiology and Intensive Care Medicine (DGAI), which aimed to develop evaluation tools for the quality of intensive care. A selection of core processes out of existing Spanish QI seemed to be adequate for this purpose [1]. The selected 10 quality indicators were thought to be generally valid for all kinds of intensive care units, and at the same time provide a framework for all professional groups working there. Since 2009, the development of intensive care quality indicators has been carried out by the National Peer Review Steering Committee (NSPR) of the German Interdisciplinary Association for Intensive Care and Emergency Medicine (DIVI). The QI and the voluntary peer review for intensive care medicine were developed as core elements of quality assurance [2], [3]. The QI developed in this process were based on the observation that there was relevant potential for improvement in intensive care processes that would have a significant effect on the quality of patient treatment. Therefore they were focused on these procedural problems. In a way, an existing problem was translated into an indicator. The achievement of a target value defined in the expert consensus, as far as possible in line with the current evidence, would correspond with good process quality, or otherwise could be used to measure improvement. The development of intensive care indicators followed a “bottom-up” principle, as they addressed practical problems perceived in daily clinical routine [2]. The rationale for a QI could thus be understood by medical professionals in intensive care directly involved in patient treatment.

This contrasts with the development of quality indicators, e.g. from treatment guidelines recommendations, in which the potential for quality improvement has to be determined beforehand (“top-down”) [4], [5], [6].

In Germany, QI exist in different clinical settings. These are developed on a national level for external quality assurance by the Institute for Quality and Transparency in Health Care (IQTIG). Through legislation such as the Hospital Structure Act and through legal guidelines for quality assurance (G-BA directive), it is expected that QI become mandatory in intensive care medicine. It is evident that quality initiatives like the QI developed under the auspices of the DIVI should be integrated into this framework of external quality assurance. Since their first publication, these QI were perceived differently over time. At present, the recording of intensive care QI is voluntary, either through voluntary peer review or through institutional quality management. However, the importance of these QI is increasing, as they have already impacted structural directives for intensive care medicine, as can be seen from their influence on intensive care reimbursement rules [7]. The demand for “evidence-based medicine” as a basis for the correct implementation of treatment processes made the development of the DIVI QI in their revisions increasingly labor-intensive, as the indicators were predominantly process indicators for which the underlying evidence frequently changes; and thus updates are required [8]. This has resulted in three revisions so far, and there is already a need for changes for the next planned revision. A comparison of the different versions of the DIVI QI is shown in Table 1 (Tab. 1). The future QI will have to meet increased demands on their own quality. Therefore, the National Steering Committee of the DIVI is planning to have the future development process methodically accompanied by the AWMF-IMWi. In order to ensure transparency in this context, this manuscript describes a potential development process and at the same time would like to induce a discussion about this revised methodology for the DIVI QI. This article explicitly does not address the medical content of single quality indicators.

## 2 Methods

### 2.1 Current development process of QI in intensive care in Germany 

The National Steering Committee Peer Review (NSPR) of the DIVI is responsible for the development of the QI. In a Delphi process, the medical societies united in the DIVI submit proposals for topics relevant to them, which are then evaluated in the NSPR. For the first development in 2010, the number of indicators was set at ten for reasons of practicability. In the development of the individual QI of the three previous versions, the criteria of the RUMBA system were applied (relevant, understandable, measurable, behaviorable, achievable) [9]. Topics were evaluated based on the following factors:

1. frequency of the process,
2. changeability of behavior,
3. magnitude of the potential for improvement,
4. (general) validity for all areas of adult intensive care.

**Figure 1:**
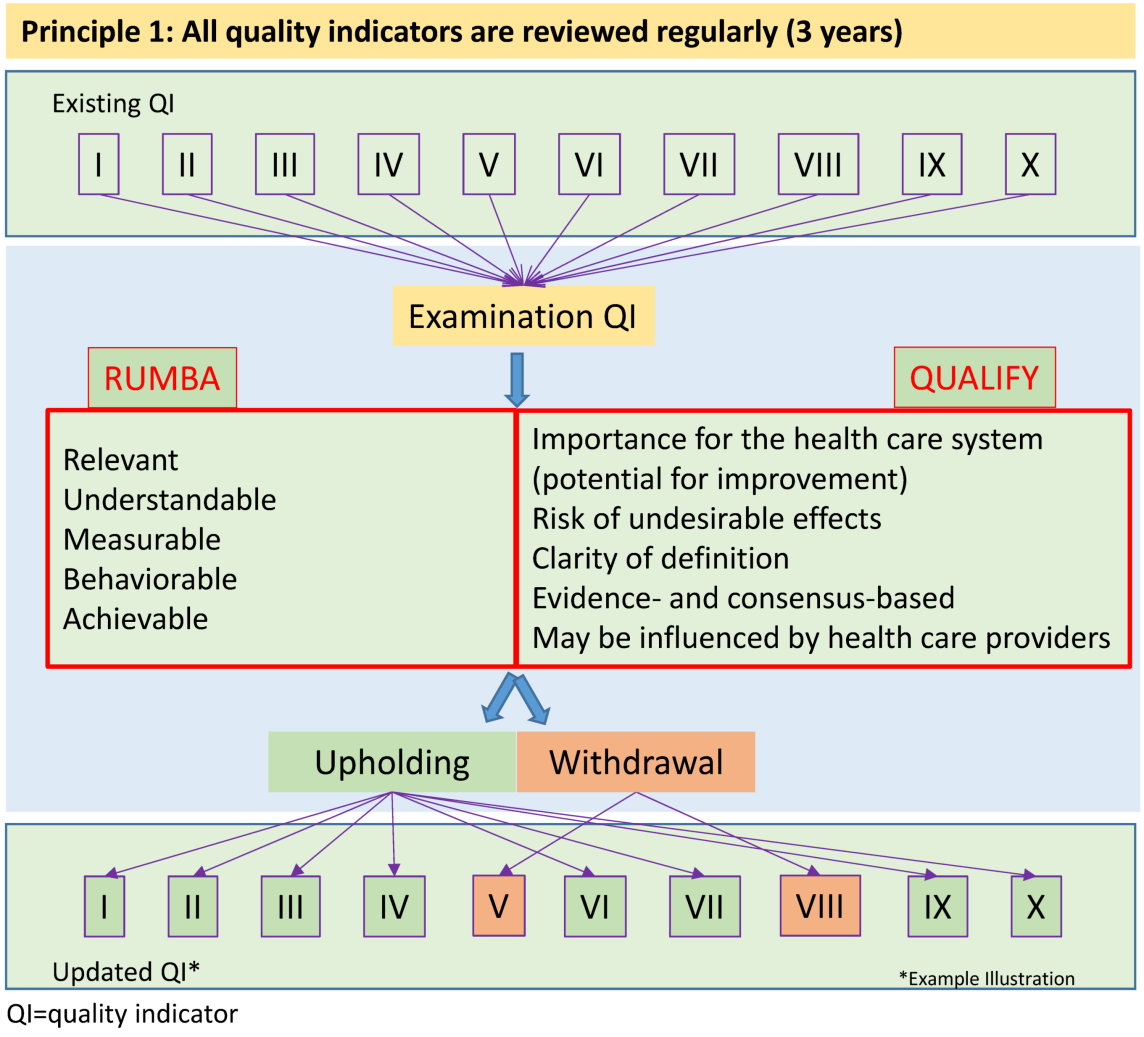
Methodology of indicator testing in a consensus-based structured evaluation process; differences between the RUMBA instrument and the QUALIFY instrument and their potential use

Another important factor was the available evidence. If applicable, the existence of external regulations had to be taken into account. Too many changes in the topics would impair the QI implementation within individual institutions, as implementation and testing of these indicators may be associated with time and personnel costs [10]. When determining the formulas for indicators and their respective target values, they were primarily based on existing values in the literature. If no data were available, a decision was made by expert consensus in the NSPR. With regard to the target value, an achievement value of 70% appeared to be reasonable on the basis of existing examples [11]. Necessary revisions or modifications of the QI were based on feedback from the voluntary peer reviews that were part of intensive care unit (ICU) evaluations. Changes resulted from direct assessments of relevance or applicability [12]. Furthermore, a potential change in the evidence basis was taken into account for a given QI. In addition, the aforementioned RUMBA rules remained the key evaluation tools. When new quality indicators were selected, the focus was mainly on the relevance of the topic in terms of the expected impact on patient outcome. The RUMBA system was also applied here (Figure 1 (Fig. 1), Table 2 (Tab. 2)). The evidence base for the indicator was then the focus of the second step, but the evidence was not formally evaluated. Possible sources of evidence were guidelines (GL), preferably valid for the German health care system and published by the AWMF – due to the structured method of their development [13]. When estimating the reliability of evidence, we assumed the best evidence from S3 guidelines, followed by recommendations from S2e guidelines and S2k guidelines. In the absence of guidelines in the AWMF register, systematic reviews or international evidence-based GL were used as a source of evidence. Finally, when no formal evidence was found, expert consensus within the steering committee was used to determine a QI based on primary literature known to the experts. As a part of the methodological framework of the DIVI peer review process, the measurement of the QI was more a random check of selective evaluations with a small number of cases (one day, one ICU) available [12], [14]. Operationalization in the sense of a quantitative survey outside this peer review process was not the primary intention of the DIVI indicators.

### 2.2 Adaptation of the QI development process 

In the continuous development process of QI in intensive care, different aspects have to be considered. On the one hand, relevant literature for QI development shows that more diligent methodological criteria should be used in the development of QI [5], [15], [16]. In addition to these methodological requirements, fundamental quality dimensions such as safety, effectiveness, patient focus, timeliness, efficiency and equity should be considered as important focal points [17]. The importance of QI in quality assurance has already been mentioned, as well as the potential for the unwelcome instrumentalization of the DIVI QI for reimbursement schemes in the DRG system (definition of intensive care treatment complexity), among other things [7]. This goes beyond a strictly medical evaluation of processes in intensive care and needs to be addressed with improved methodological quality in their development. The development process used to date has been described in the publications on DIVI QI [18], [19]. The further development is based on various relevant publications, i.e. existing manuals for guideline development [4], [5] for German-speaking countries as well as the QUALIFY evaluation tool developed in recent years [20]. These serve as the basis of criteria used in guideline manuals. This method of the future development process of the DIVI QI thus includes an explicit scientific evaluation process and also takes into account requirements for quality thesholds [6], [16]. Similar to the development process of guidelines, the disclosure of conflicts of interest is also relevant in the process of QI development.

In the following sections, the steps for the development of the next edition of QI, which will be published in 2021, are described and presented for discussion. The evaluation of existing indicators, the development of new indicators including pilot testing, the definition of suitable key figures, and the cooperation in the development process will be addressed.

### 2.3 Evaluation of the previous QI 

As a first step in the development of the next version of intensive care QI, evaluation criteria were defined, which are based on the QI evaluation instrument QUALIFY [20]. They include the following aspects:

1. importance for the health care system/potential for improvement,
2. risks of undesirable effects,
3. clarity of definition,
4. evidence- and consensus-based (in guidelines),
5. potential to be influenced by health care providers.

These rules differ from the RUMBA system used to date (Table 2 (Tab. 2)). The comparison with the QUALIFY criteria and the criteria modified for guideline development show that factors beyond applicability have to be taken into account. A suitable evidence base for indicator development is needed. Ideally, the use of the indicator itself has a positive effect on patient outcome [21], [22].

As described in section 2.1, the methodological basis for indicator modification was not rigidly defined. By applying the criteria listed in Table 2 (Tab. 2), a systematic approach has to be developed which allows a direct comparison of QI for a particular topic, or further assessment of QI results over longer periods of time [20]. The review of the QI may lead to their confirmation, modification or withdrawal. Modifications may also affect the reference value or reference range of the indicator measure. The QUALIFY instrument is also suitable for making changes of a QI or its withdrawal transparent and comprehensible, e.g. if the evidence basis is not consistent or an alternative indicator is available for a specific topic [5].

### 2.4 Development of new indicators

**Figure 2:**
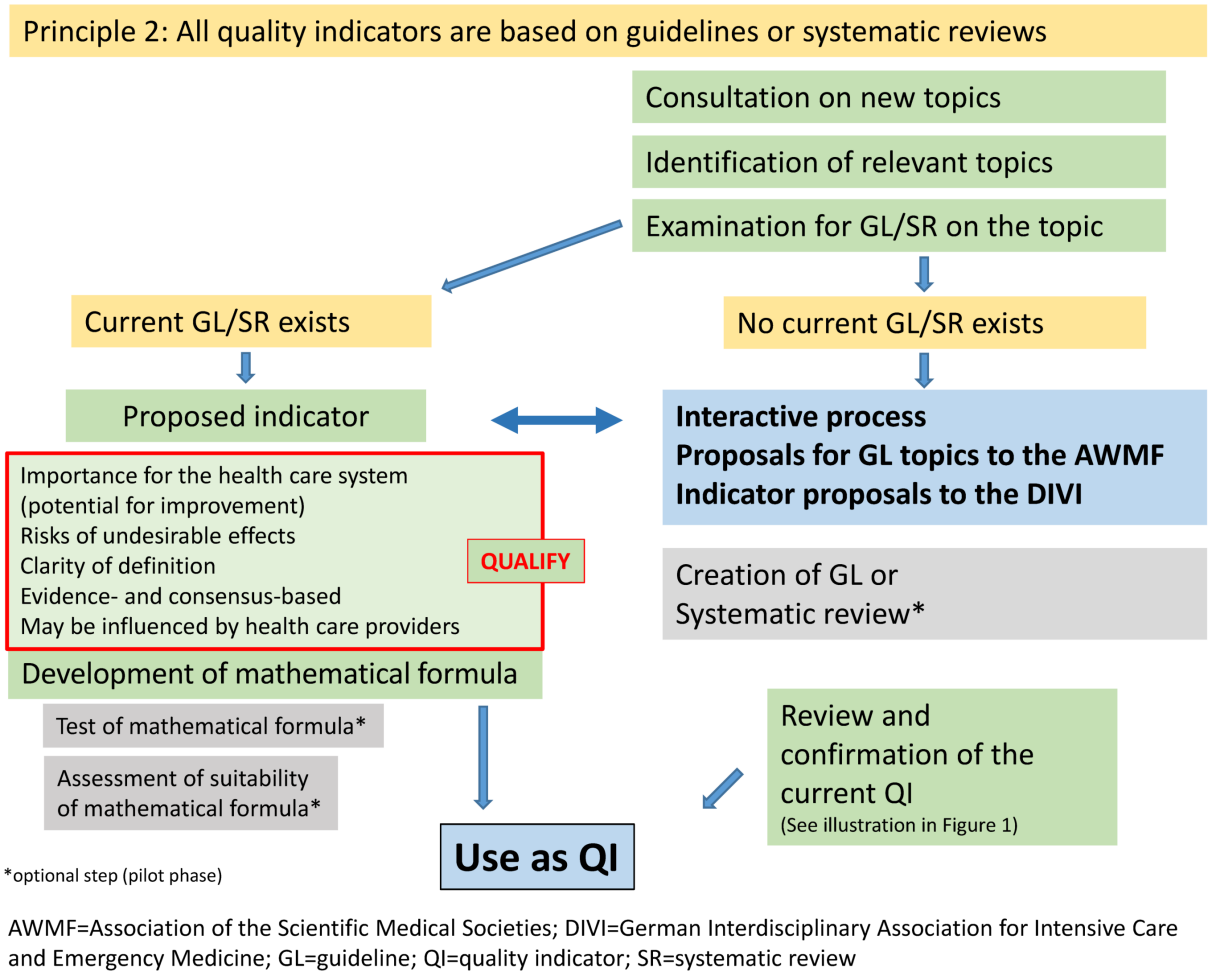
Methodological approach for further development of intensive care quality indicators on the basis of existing guidelines; structured evaluation process using the modified QUALIFY system

First a review is carried out in the NSPR on the basis of proposals for new indicator topics from the medical societies that are members of the DIVI. Figure 1 (Fig. 1) and Figure 2 (Fig. 2) show the steps of the indicator development. One important change is the focus on evidence-based criteria derived from the QUALIFY instrument. As shown in Table 2 (Tab. 2), there is a clear difference between the previous method using the RUMBA system and the new one. The new, more systematic evidence research will also include estimates of potential for improvement of a treatment process on patient outcome. We postulate that if there is a high level of evidence for process adherence and a demonstrable positive outcome, the level of indicator evidence is high (provided that the positive patient outcome is the endpoint of the reviewed evidence). The available evidence is assessed as described above (guidelines, systematic reviews, meaningful primary literature) and largely follows the GRADE system (Grading of Recommendations Assessment, Development and Evaluation) [23], [24].

Based on the strength of the evidence, the indicator strength will mainly be determined by consensus. This makes sense, since a high level of indicator evidence may still be available if there is an obvious correlation between process fulfilment and outcome (e.g. if no direct studies are possible) [20].

### 2.5 Pilot testing

It is desirable that the aspects mentioned in sections 2.3 and 2.4 are pilot tested before a newly developed QI is generally applied. The test focuses on acceptance (by the user) and methodological suitability, including proof of validity, statistical aspects and indicator evidence [8]. The statistical requirements such as ability to discriminate between “good” and “poor” practice and sensitivity to change are discussed below. Due to the previous use of QI in the peer review process of the DIVI, the development of rate-based QI with numerator and denominator and adaption of calculation rules if required as well as statistical aspects have so far been secondary. Quantitative or qualitative assessments of individual indicators should be carried out (Figure 2 (Fig. 2)) for that purpose. This is also necessary to check quality requirements for indicator function, i.e. relevance for outcome measures. Pilot testing can be obtained from existing data records. This is further explained in the following section.

### 2.6 Further development and use of suitable key figures

The definition of key figures and reference values has so far been based on expert consensus within the NSPR and was based on the existing literature or known indicators. Reference ranges (upper and lower limit values) were determined in a similar way. In the future, determination of indicator key figures and their limits is a central aspect for the development and use of indicators. Two aspects will be essential in this context:

1. collection of valid data to be able to calculate indicators,
2. evaluation of the indicators in relation to the established reference ranges.

As mentioned in the previous section, these questions are of particular importance when assessing QI in test phases. As shown in Table 2 (Tab. 2), comprehensibly collected data are important in the implementation and application of QI in order to strengthen the credibility of the measurement results, e.g. within an institution [10], [16]. When the indicators are applied across institutions, further requirements such as content validity are added [6], [25]. Easily measurable indicators would facilitate their application in settings outside a peer review process. The automated collection of data for the calculation of key figures would be an important step in initiating and evaluating quality improvement measures within an institution and in quality assurance. Of note, the development of such key figures is also challenging from a statistical point of view, as it is difficult to verify accuracy or the scale level due to scarcely available empirical data. Therefore it is critical to show validity of measurement characteristics by pilot testing, and QI results have to be interpreted by methodological and clinical experts.

### 2.7 Cooperation in indicator development

In the future, the evaluated indicators should be actively shared with guideline development groups working on intensive care topics (Figure 2 (Fig. 2)). This should enable a faster and more efficient definition of the quality indicators for the use in guidelines relevant to intensive care medicine. In cooperation with the guideline developers under the auspices of the AWMF-IMWi, these QI will be evaluated and, if necessary, combined with GL recommendations. The adapted QUALIFY criteria will already be applied in the guideline groups. In the future, guideline developers will be able to share relevant new evidence or derived GL recommendations to the NSPR of the DIVI. Furthermore, new GL recommendations that are based on new evidence and might lead to new indicators or new topics relevant for QI development might be shared with the NSPR. Overall, this exchange between the NSPR and guideline development groups will result in synergy effects. It results in synergy effects which both sides will benefit from in the future. The guideline development groups should also examine the extent to which health care data generated by the use of the established indicators can be used in the development and evaluation of future indicators.

## 3 Results

The development and use of quality indicators aims to improve treatment processes and patient outcome. The QI for intensive care medicine of the DIVI meet these requirements. However, as is presented in this paper, there is potential for improvement in the development of the QI in some areas. These relate to the evidence basis of the QI at the patient level and the health care system level and also their definite operationalization to enable broad application. A considerable number of intensive care QI exist worldwide [26]. Their most common feature is to evaluate important treatment processes in intensive care. However, there are no methodological standards for their development or their application [27]. One main reason for this are context factors which have be taken into account when applying QI. This also applies to the intensive care quality indicators of DIVI, which were developed from the voluntary intensive care peer review process in order to identify potential for improvement in treatment processes on single sites in Germany.

### 3.1 Evidence-based approach

The postulated “lack of evidence basis” of QI mentioned in this paper is not intended to discredit the QI developed so far, but to emphasize that the transfer of guideline recommendations into QI is a very complex issue and should therefore be done in a structured and comprehensible way. The following general rule applies: The higher the published evidence for the implementation of a treatment process, the stronger is the evidence for the use of an indicator that evaluates this process. This basically refers to the strength of evidence, especially of guideline recommendations, where this basic principle is based on evaluation criteria from the GRADE system such as inaccuracy, indirectness and inconsistency, as well as others. The evaluation of GL according to the GRADE system cannot be applied directly to QI. However, the described assessment criteria for the transferability of study results into actual treatment recommendations can have an influence on the QI, since GL are the basis of the QI as described, and problems with study safety (=certainty) affect the QI [28], [29]. It may be possible that studies are not feasible for certain aspects of care, in which case the indicator may still be useful and effective [15]. The selection of indicator topics for intensive care QI has not been based on the best evidence yet. It followed the approach of the DIVI NSPR described above on the basis of perceived quality deficits in daily patient care. A combination of the two strategies seems to make sense. In order to improve transparency and traceability of the development strategy, this must be justified and documented in a structured way as described by the QUALIFY method. There are various examples of the application of the QUALIFY method in the German-speaking countries for the preparation and assessment of QI [30], [31].

### 3.2 Operationalization 

In Germany, the possibility of standardized data collection, ideally in a digital format, is not available across the country. For this reason, local measurability must currently be considered when developing indicators. The process indicators of the DIVI are well suited for this. However, in order to meet important statistical requirements for a broader application of QI, it is necessary to obtain sufficient data volumes. Here, the opportunities offered by digitalization must be used consistently. In a next step, questions of the statistical evaluability have to be focused on: the question of content validity – that the QI actually measures what it is intended for –, the ability to discriminate between institutions, and the sensitivity to measure change over time, e.g. to detect improvements or failure to improve. Finally, the data have to be consistent enough to evaluate an outcome effect of the indicator in use, since this effect should be as large as possible. The sufficient amount of data can only be collected through the broad implementation of patient data management systems (PDMS) in all ICUs. This would enable low-cost data collection and cross-sectoral data collection on patient-centred outcome parameters [7], [32]. However, as long as the technical prerequisites for recording these data are not in place, intersectoral indicators cannot be measured either [33]. In the long term, a comparison of institutions also on the basis of process indicators is a worthwhile goal. It seems reasonable to strive for these comparisons as well, if possible.

### 3.3 Outcome benefits of quality indicators

When a QI is implemented, it is necessary to evaluate whether the chosen indicator is reliable and valid, and whether its measurement can initiate a process of change within an institution or system, and how this is presented. Benefit assessments of QI have to state whether the conclusions drawn from the QI evaluation are reliable enough to prove a change in processes. For the use of QI – at least in intensive care medicine – a positive influence through the use of indicators on patient outcome has not been proven yet. It is also not known if QI can have a negative impact on patient outcome. For example, the application of sedation pauses, as is recommended, might increase the rate of self-extubation, which in itself might pose a risk to the patient. Valid data on this question do not exist. Therefore, a formal risk assessment should be integrated into the QI development process whenever possible. If necessary, an indicator must be withdrawn or amended in such a case.

The type of indicator used also plays a role in the benefit assessment. In intensive care, there are some arguments in favor of using process indicators, as they are easy to measure and do not require risk adjustment for disease severity. However, in the literature some indicators are referred to as “process indicators” even if they query a result, e.g. rate of ventilator-associated pneumonia (VAP) as an indicator of appropriate hand hygiene [27]. This would mean that the VAP rate is exclusively or substantially influenced by the process hygiene behavior, which might not be true [34]. There is also a difference in measurement of process adherence, i.e. the process itself is recorded (example: regular measurement of a delirium score), or a key figure for process adherence (here: number of measurements in a defined period of time) is measured. No formal assessment of this has been examined for an effect on patient outcome. The need to prove an outcome benefit of process indicators should be examined carefully. If the underlying evidence shows a direct and strong correlation of process and outcome, the effort to show this benefit might not be necessary. Otherwise, to proof the usefulness of a process QI with regard to outcome is relevant, and there should be a scientific concept of how to define and measure this outcome effect.

For the quality dimensions proposed by Donabedian, not only process indicators are considered useful to evaluate a quality problem, but also structure and outcome indicators [35]. In the DIVI QI, the existence of a process standard, i.e. a structure indicator, may be required in association with a process indicator. The definition of evidence-based structural indicators is certainly difficult in most cases, as they are often based on experience or tradition, rather than on comparative studies. They can sometimes only be defined by expert consensus [36]. In principle, a process can be performed well even without a standard in place. A process evaluation would then indicate whether standards are followed in daily routine. Outcome indicators are used, for example, in cross-institutional benchmarking. One problem of their application is the need for risk adjustment due to heterogeneous patient groups. Outcome indicators are also only of limited value when evaluating the implementation of a process. In case of a deviation from the reference value of the indicator, processes have to be examined in retrospect. Nevertheless, comparisons of institutions tend to use outcome indicators due to better data availability. Other outcome indicators, such as mortality – even if they are standardized for risk factors (SMR) – are not able to record actual process adherence [37]. In addition, such indicators are at best only able to measure undesirable developments after longer periods of time (e.g. months), which may be due to incorrectly implemented processes or the failure to meet structural requirements [38]. However, the measure is slow and may tend to miss relevant quality problems.

## 4 Conclusion

The following statement by Califf et al. represents an “ideal state” in quality management and quality assurance: “In an ideal clinical world, for every clinical decision there would be an indicator based on a guideline based on evidence from randomized trials” [39]. It is unlikely that the costs and benefits of this approach can be reconciled. However, the sentence confirms that clinical decisions in a process-based approach can produce a positive treatment outcome in the patients’ interest. This is the basic principle of the QI in intensive care of the DIVI. The methodology for the future development of intensive care QI presented here shows that these indicators will have a robust basis for use within a single institution as well as for other applications. The planned synergistic approach through a cooperation with AWMF guideline groups and the use of the criteria from the QUALIFY instrument should further strengthen the scientific basis of QI. The coordinated development process is intended to simplify the work of the NSPR of the DIVI, as well as of the respective guideline development groups working according to the AWMF-IMWi rules, especially by avoiding redundant development steps such as literature research and development of the key figures. In particular, the use of guideline-based QI should bring the patient perspective to the forefront of indicator development. This will be one of the biggest challenges in the future [15]. The approach described also makes the intensive care indicators suitable for their use beyond single institutions, e.g. in external quality assurance. This would ensure that evaluation of intensive care treatment processes will remain under the control of their providers.

## Abbreviations

- AWMF: Association of the Scientific Medical Societies
- DIVI: German Interdisciplinary Association for Intensive Care and Emergency Medicine
- GBA: Federal Joint Committee
- GL: guideline
- GRADE: Grading of Recommendations Assessment, Development and Evaluation
- IQTIG: Institute for Quality Assurance and Transparency in Health Care
- NSPR: National Steering Committee Peer Review
- NVL: national disease management guideline
- QI: quality indicator
- QS: quality assurance
- VAP: ventilator-associated pneumonia

## Notes

### Competing interests

The authors declare that they have no competing interests.

## Figures and Tables

**Table 1:**
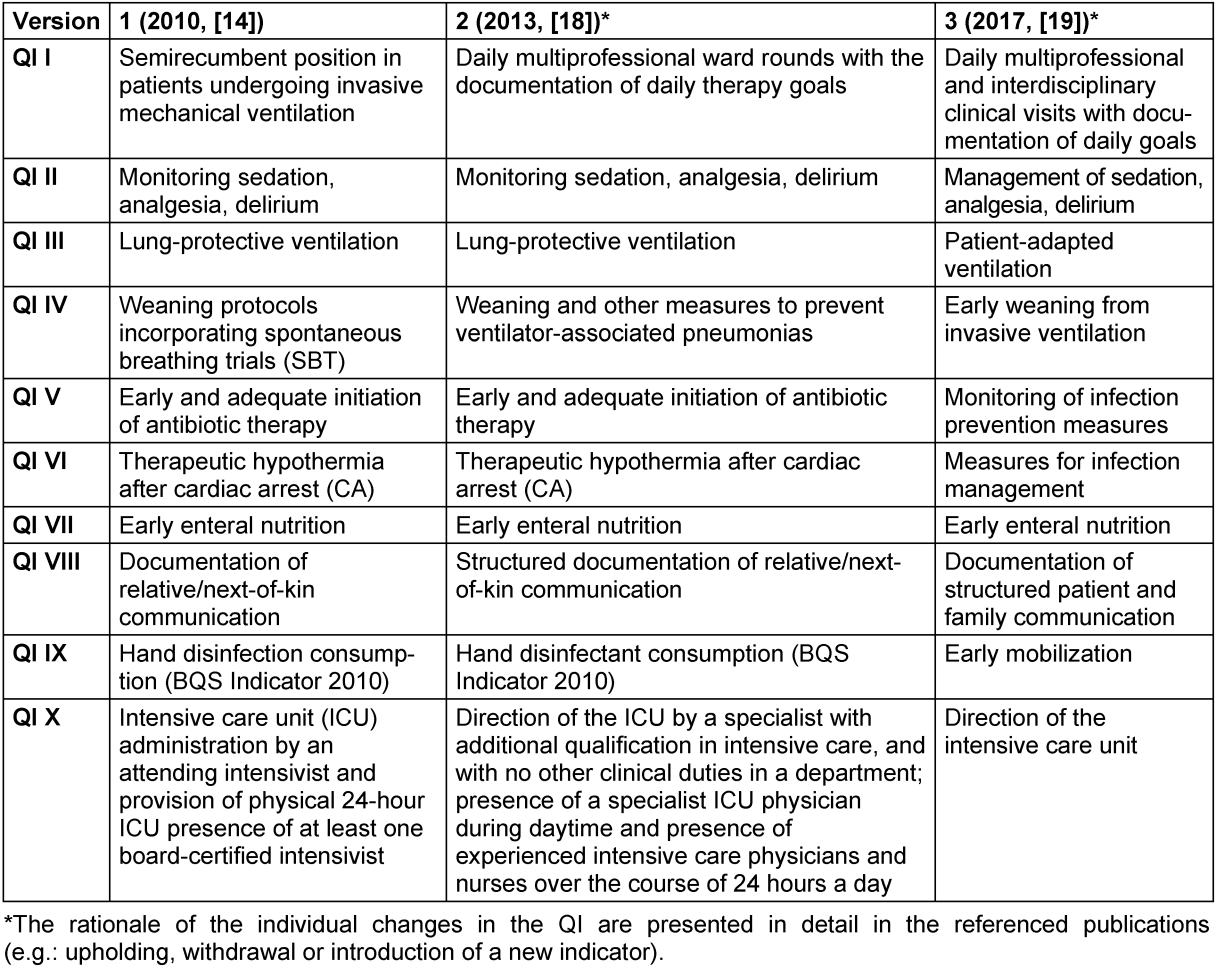
Comparison of different versions of the DIVI QI [14], [18], [19]

**Table 2:**
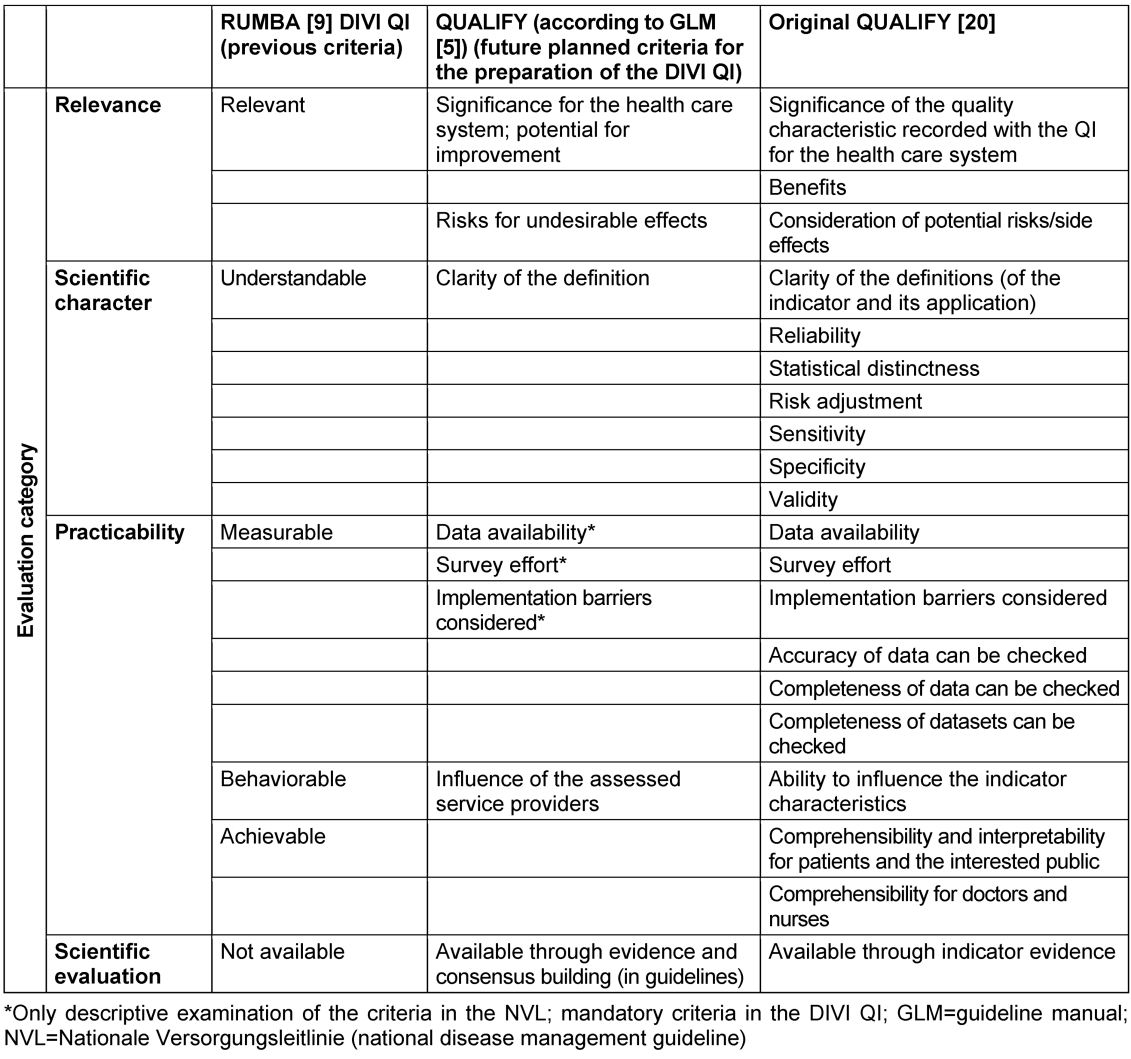
Comparison of evaluation criteria for quality indicators (RUMBA/QUALIFY criteria for guideline-based QI/original QUALIFY)
